# Quinacrine Has Preferential Anticancer Effects on Mesothelioma Cells With Inactivating NF2 Mutations

**DOI:** 10.3389/fphar.2021.750352

**Published:** 2021-09-21

**Authors:** Derek B. Oien, Sayantani Sarkar Bhattacharya, Jeremy Chien, Julian Molina, Viji Shridhar

**Affiliations:** ^1^ Division of Experimental Pathology and Laboratory Medicine, Mayo Clinic, Rochester, MN, United States; ^2^ Department of Biochemistry and Molecular Medicine, University of California, Davis Health, Sacramento, CA, United States; ^3^ Department of Medical Oncology, Mayo Clinic, Rochester, MN, United States

**Keywords:** quinacrine, mesothelioma, NF2, merlin, YAP, hippo signaling, repurposed drug

## Abstract

Mesothelioma is a rare cancer with disproportionately higher death rates for shipping and mining populations. These patients have few treatment options, which can be partially attributed to limited chemotherapy responses for tumors. We initially hypothesized that quinacrine could be combined with cisplatin or pemetrexed to synergistically eliminate mesothelioma cells. The combination with cisplatin resulted in synergistic cell death and the combination with pemetrexed was not synergistic, although novel artificially-generated pemetrexed-resistant cells were more sensitive to quinacrine. Unexpectedly, we discovered cells with NF2 mutations were very sensitive to quinacrine. This change of quinacrine sensitivity was confirmed by NF2 ectopic expression and knockdown in NF2 mutant and wildtype cell lines, respectively. There are few common mutations in mesothelioma and inactivating NF2 mutations are present in up to 60% of these tumors. We found quinacrine alters the expression of over 3000 genes in NF2-mutated cells that were significantly different than quinacrine-induced changes in NF2 wildtype cells. Changes to NF2/hippo pathway biomarkers were validated at the mRNA and protein levels. Additionally, quinacrine induces a G1 phase cell cycle arrest in NF2-mutated cells versus the S phase arrest in NF2-wildtype cells. This study suggests quinacrine may have repurposing potential for a large subset of mesothelioma patients.

## Introduction

Malignant pleural mesothelioma is cancer arising from the mesothelial cells of the pleural cavity serosal lining. These tumors are caused by asbestos exposure and more recently have been associated with taconite mining in Minnesota (Mandel and Odo, 2018). Mesothelioma is often diagnosed at advanced stages when surgical options are very limited. These tumors are notoriously treatment-refractory. The median overall survival for standard radiation and chemotherapy is about 12 months (Carbone et al., 2019), and 18 months for recently approved immunotherapies. To address the lack of response for tumors to standard cisplatin and pemetrexed chemotherapy, we had previously investigated adjuvant targeted drugs in mesothelioma (Oien et al., 2017). Based on recent developments for using quinacrine in treatment-refractory cancer (Oien et al., 2019), we hypothesized that quinacrine may be effective against mesothelioma cells.

Quinacrine was rediscovered as an anticancer agent in two separate screens. Quinacrine was found to induce p53 in a small molecule screen using renal carcinoma cells with minimal basal wildtype p53 protein expression (Gurova et al., 2005). It was later shown that quinacrine is cytotoxic in cells with TP53 mutations, including studies from our laboratory (Khurana et al., 2015; Kalogera et al., 2017; Jung et al., 2018). In a separate small molecule library screen, quinacrine was found to be active against leukemia patient samples spanning three subtypes with concurrent minimal toxicity in non-malignant peripheral blood mononuclear cells (Eriksson et al., 2015). This latter study highlights the proposed selective toxicity of quinacrine towards cancer cells (Gurova, 2009). Quinacrine was historically used for antimalarial prophylaxis/treatment and is still used in developing countries for female sterilization with minimal long-term side effects (Oien et al., 2019). Quinacrine is believed to have polypharmacology characteristics impacting p53-associated apoptosis, autophagy, ribosomal biogenesis, and PRMT5 downregulation (Busacca et al., 2021), yet no predominant anticancer mechanism has been identified. However, the tumor response to quinacrine in clinical trials so far has not been impressive (Bhateja et al., 2018), and this underlies the importance of finding tumors that will have a response to quinacrine treatment or effective drug combinations with quinacrine.

We previously reported that quinacrine can resensitize drug-resistant gynecologic cancer cells to cisplatin, carboplatin, and paclitaxel (Khurana et al., 2015; Kalogera et al., 2017). This supported our initial hypothesis that drug-resistant mesothelioma cells will be more sensitive to quinacrine, and combining quinacrine with cisplatin or pemetrexed will enhance cytotoxicity. However, we noticed that NF2-mutated cells were also more sensitive to quinacrine compared to NF2 wildtype mesothelial, lung, ovarian, and breast cancer cells.

NF2 (neurofibromin 2 protein, aka merlin) is a tumor suppressor that controls cell division and cellular contact inhibition through the hippo pathway, and inactivating NF2 mutations drive tumor growth through a loss of control in hippo signaling (Sato and Sekido, 2018). The *NF2* gene is frequently mutated in mesothelioma, ∼40% in TCGA samples and 40–60% in the literature (Sato and Sekido, 2018), which is uncommon for most other cancer types. However, there are no treatments to specifically address tumors with *NF2* genetic alterations (Oien et al., 2020). In this study, we used RNA sequencing to discover quinacrine modulates NF2/hippo signaling pathways.

## Materials and Methods

### Cell Lines and Drugs

Human malignant pleural mesothelioma cell lines NCI-H28, NCI-H226, NCI-H2052, and HCI-H2452 (Sarkar Bhattacharya et al., 2019), and non-small cell lung cancer cell line A549 were obtained from ATCC. Human malignant pleural mesothelioma H2591 cells were generated by and obtained from the Harvey Pass laboratory (Pass et al., 1995). The murine malignant mesothelioma AE17 cell line (Sigma) is a model derived from the peritoneal cavity of C57BL/6J mice injected with crocidolite asbestos fibers (Jackaman et al., 2003). The cell line status for common genetic alterations in mesothelioma is described in Supplementary Table 1. All cell lines were cultured in Gibco RPMI-1640 media with 10% FBS (ThermoFisher). Medium for virus-transformed human mesothelium cell line Met-5A cells was supplemented with epidermal growth factor (3.3 nM), hydrocortisone (400 nM), zinc-free bovine insulin (870 nM), and HEPES (20 mM) (Oien et al., 2017). Cisplatin USP (Teva) was supplied as a 1 g/L solution in saline, and pemetrexed (Sigma) was dissolved in water immediately prior to use. Quinacrine dihydrochloride (Sigma) was stored as 5 mM aliquots in water at −80 C.

### Generation of Cisplatin and Pemetrexed-Resistant Cell Lines

Artificial cell line resistance was generated by incremental cisplatin or pemetrexed dosing of cell lines for up to 4 months. Drug was added to cultured cells for 3 days until 10–20% of viable cells remained followed by a 4 days drug holiday for regrowth as a weekly cycle. For the next cycle, drug doses were incremented from 0–50% based on the cell death and regrowth characteristics of the prior week. Interim resistance was monitored by cell death assays. To avoid mistaking reversible drug tolerant persister cell phenomena (Sharma et al., 2010) for irreversible drug resistance, parallel cell cultures were taken from weekly cycles and maintained for at least 2 weeks without drug before subjecting to clonogenic assays for verification or other drug response assays.

### Cell Death Assays and Drug Synergy Calculations

Short-term cell death assays and synergy calculations were performed as previously described (Bastola et al., 2016; Oien et al., 2017). Briefly, 3,000 cells/well were seeded in 96-well plates and attached by overnight incubation prior to indicated drug treatment. After 72 h, cells were fixed with 10% trichloroacetic acid (Sigma). Fixed cells were washed with cold water and stained using sulforhodamine B (SRB, Sigma) at room temperature. Cells were washed with 1% acetic acid solution and then were dissolved in 10 mM Tris, pH 10, and fluorescence measurements (488/585 nm excitation/emission) were taken using a Synergy4 plate reader (BioTek). Where specified, the 50% inhibition concentration (IC_50_) was estimated using Prism software (GraphPad). Drug synergy was determined by calculating the combination indices (CIs) obtained from the fluorescence measurements. The CI values were calculated based on dividing the combination expected effect by the observed effect: CI = [D1 +D2 *(1-D1)]/D_observed_, where D represents the cell death from drug 1, drug 2, and the combination (observed) (Chou, 2010). The mean CI values were determined from 15 drug combinations (5 cisplatin and 3 quinacrine concentrations) in duplicates for each cell line.

Long-term clonogenic assays were performed as previously described (Bastola et al., 2016; Oien et al., 2017). Briefly, 500 cells/well were plated in 6-well plates and cells were attached by overnight incubation. Regents were added to cells and incubated for 72 h. Following treatment, medium was gently aspirated and replaced with regular growth medium. Cells were then incubated for four to seven additional days. Once the colonies were optimal for visualization, cells were fixed were fixed with 10% trichloroacetic acid and stained with SRB. Prior to quantification, plates were air-dried, and pictures were taken using a Gel Doc imager (Bio-Rad).

### NF2 Plasmid Expression and siRNA Knockdown

Ectopic NF2 expression was performed by plasmid electroporation. Plasmid was obtained as glycerol stock [pEGFP-merlin #84293, Addgene (Xiao et al., 2002)], inoculated in LB broth plus kanamycin, and isolated with Maxi EF kit (Qiagen). Briefly, 5.3 µg *NF2* or empty vector plasmid was added to 600,000 cells in OptiMEM (ThermoFisher) medium. Cells and plasmid were added to a cuvette and pulsed in Gene Pulser Xcell ShockPod (Bio-Rad) using a 150V square wave for 15 ms. Cells were seeded in a culture dish for 24 h with G418 (Invitrogen) selection prior to indicated treatment.

*NF2* siRNA knockdown was performed by lipid-based transfection. About 4 µL (4 × 10^−11^ mol) siRNA (sc-36052, Santa Cruz) or NTC was combined with P3000 (1:5 plasmid:P3000, Invitrogen) and Lipofectamine 3000 (6 times plasmid volume, Invitrogen) in Gibco OptiMEM medium (ThermoFisher). The mixture was briefly incubated and added to cells for 6 h followed by regular medium for 24 h prior to indicated treatment.

### Immunoblots and Subcellular Fractionation

Immunoblot protein lysates were produced as previously described (Oien et al., 2017). Briefly, cells were seeded in 6-well plates and attached by overnight incubation. Reagents were added to cells and incubated for 72 h. Cells were washed with PBS and collected in Laemmli sample buffer (Bio-Rad) with protease/phosphatase inhibitors and reducing agent (Sigma) by scraping. Samples were sonicated and heated to 95 C prior to loading in TGX gels (Bio-Rad). Proteins were transferred to Immobilon FL membranes (Millipore) and blocked in buffer (Li-Cor) overnight. All primary antibodies were incubated overnight and visualized by an Odyssey Fc Imaging System (Li-Cor). Primary antibodies were merlin/NF2, YAP1, GNL3/nucleostemin, p53 from Santa Cruz and GAPDH, cyclin D1, CDK4, PARP, cleaved PARP from Cell Signaling.

Subcellular fractionation was achieved by subjecting cell pellets to buffer containing 10 mM HEPES, 50 mM NaCl, and 500 mM sucrose with 0.7% TritonX-100 (Sigma) and centrifuging at 1000 x g to obtain the cytoplasmic fraction. The remaining cell pellet was washed with harvest buffer and centrifuged at 2000 x g, discarding the supernatant to leave a pellet of the nuclear fraction. Subcellular fractionation was verified by α-tubulin and H3 antibodies (Cell Signaling) (Kumar et al., 2019).

### RNA Sequencing

Cells were treated with 5 µM quinacrine for 6 h followed by RNA isolation with TRIzol (Ambion)/chloroform (Sigma). Crude RNA was purified with RNeasy columns (Qiagen). Sequencing was performed by Novogene Corporation (Sacremento, CA). TopHat (Trapnell et al., 2012) was used to align reads to the Human Reference Genome (hg19), and HTSeq (Anders et al., 2015) was used to produce read counts. A heatmap was generated with Morpheus software (Broad Institute). Gene analysis was performed with BRB Arraytools (Simon et al., 2007), DESeq2 (Love et al., 2014), and Ballgown pairwise comparison to identify differentially expressed genes with a *p*-value of <0.05. A Venn map was generated by Venny 2.0 online software (bioinfogp.cnb.csic.es/tools/venny/index.htm).

Gene lists for differential expression were evaluated by the Enrichr gene list enrichment analysis tool to identify similarity to KEGG 2021 pathways (Kanehisa et al., 2021) for genes perturbed by quinacrine. The two comparisons were: 1) quinacrine-induced expression changes of H2591 and H2052 compared to vehicle (303 genes, 20 genes in common with H2452 and H28 were excluded), and 2) quinacrine-induced expression changes of H2591 and H2052 compared to quinacrine-induced expression changes of H2452 and H28 (3168 genes).

### Real-Time Quantitative PCR

Expression of selected genes were validated as previously described (Jung et al., 2018). Briefly, 1 µg RNA was reverse transcribed using Quantifect cDNA Synthesis kit (Qiagen). Resulting cDNA was added to SYBR-Green PCR Master Mix (Applied Biosystems) and primers (Integrated DNA Technologies, Supplementary Table 2). Reactions were executed using a CFX96 Real-Time PCR system (Bio-Rad) and normalization across samples was by comparison to RPLP0 expression.

### Cell Cycle and Reactive Oxygen Species Analyses

Cell cycle analysis was performed as previously described (Sarkar Bhattacharya et al., 2019). Briefly, cells were dosed as indicated for 24 h. Cells were harvested by trypsin, washed in PBS with 1% BSA, and fixed in 70% cold ethanol. Pellets were treated with RNase A (Qiagen) and permeabilized with 0.1% TritonX-100 (Sigma). Cells were stained with propidium iodine (40 µg/ml final, Sigma) for 30 m at room temperature. Cells were analyzed using a FACSCalibur flow cytometer (Mayo Flow Cytometry Facility) with a minimum of 20,000 events and distribution percentage was calculated with CellQuest Pro software (BD Bioscience).

Total reactive oxygen species were measured by fluorescence detection using the ROS-ID Total ROS detection kit (Enzo Life Sciences) per manufacturer’s instructions. Briefly, 2,500 cells/well were seeded in a black 96-well plate and incubated overnight for attachment. Cells were dosed with quinacrine as indicated for 4 h. After dosing, cells were washed with PBS and ROS-ID dye was added for 30 m. Fluorescence was detected (488/520 nm excitation/emission) in a Synergy4 plate reader. Unstained and 0.5 mM hydrogen peroxide were used for negative and positive controls, respectively.

### Statistics

All results were expressed as a mean with standard error and obtained from at least three separate experiments. All statistical analyses were performed using Prism 7.05 (GraphPad) software for non-linear regression or t-test or one-way ANOVA as appropriate except when other software was noted. *p* values >0.05 were considered non-significant.

## Results

### Quinacrine Response in Chemotherapy-Resistant Cell Lines and in Combination With Chemotherapy

The response of pleural mesothelioma to pemetrexed and cisplatin treatment is short-lived with a median time to progression of 5.7 months (Vogelzang et al., 2003). Mesothelioma cell lines also have limited sensitivity to cisplatin and pemetrexed treatment (Oien et al., 2017). We had previously investigated quinacrine in combination with platinum drugs and as a monotherapy for isogenic paired gynecological cancer cells (Khurana et al., 2015; Kalogera et al., 2017). Based on the results of our past studies, we tested quinacrine in mesothelioma with artificially generated drug-resistant cell lines and in combination with cisplatin and pemetrexed.

#### Generation of Pemetrexed- and Cisplatin-Resistant Cell Lines

**FIGURE 1:**
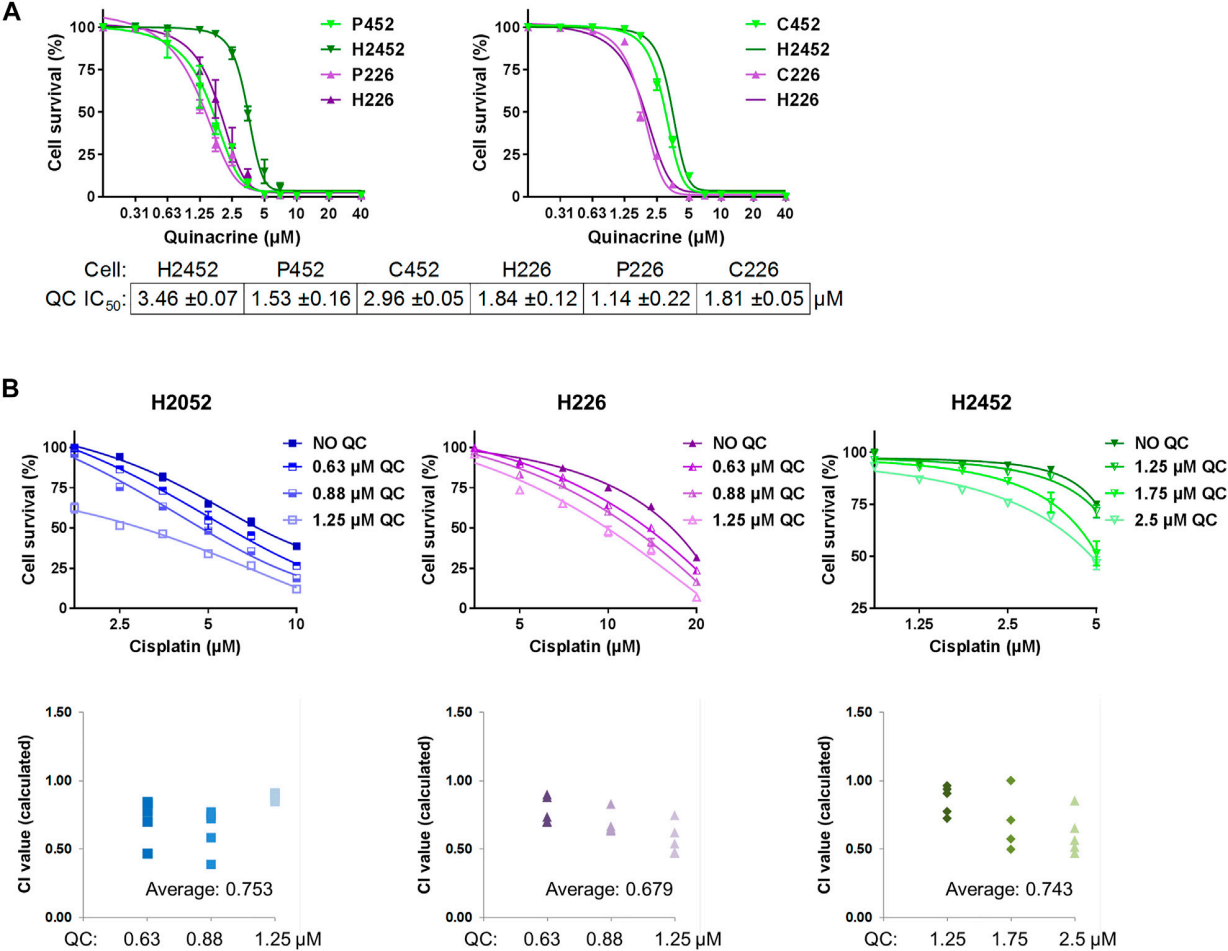
Quinacrine response compared with isogenic drug resistant cells and in combination with cisplatin. **(A)** Cell death assays using quinacrine in pemetrexed resistant (P452, P226) and cisplatin resistant (C452, C226) cells compared to isogenic controls (H2452 and H226). All 6 cell lines were assessed in parallel and curves for H2452 and H226 are shown in both graphs (the second time omitting error bars). **(B)** Cell death assays using cisplatin with different concentrations of quinacrine. Combination index values (*bottom*) were calculated for each combination. Values below 1.00 are considered synergistic.

Drug resistant cell lines were generated by cycles with incremental doses of pemetrexed or cisplatin. The H2452 and murine AE17 cell lines initially responded to both pemetrexed and cisplatin, and the H226 cell line was used for comparison because it was previously reported as insensitive to pemetrexed (Iwahori et al., 2013). Decreases in pemetrexed and cisplatin sensitivity were achieved for all cell lines as determined by final evaluation using clonogenic assays after being cultured without drug for at least 2 weeks (Supplementary Figures 1 and 2). For pemetrexed GI_50_ values, resistant H2452 “P452” cells had a final GI_50_ of 3.12 ± 0.14 µM compared to 0.94 ± 0.02 µM for H2452 (3.3-fold change) and resistant H226 “P226” cells had a final GI_50_ of 13.89 ± 0.43 µM compared to 3.96 ± 0.24 µM for H226 (3.5-fold change, Figure 1A). For cisplatin GI_50_ values, resistant H2452 “C452” cells had a final GI_50_ of 3.64 ± 0.06 µM compared to 0.91 ± 0.28 µM for H2452 (4.0-fold change) and resistant H226 “C226” cells had a final GI_50_ of 4.66 ± 0.12 µM compared to 2.11 ± 0.05 µM for H226 (2.2-fold change, Figure 1B). Overall, these resistant cells lines maintained a range of 2.2–4.0-fold decrease in susceptibility from the original cell lines.

#### Pemetrexed-Resistant Cells Are More Sensitive to Quinacrine and Quinacrine Is Synergistic With Cisplatin

After the generation of drug-resistant cell lines for H2452 and H226, quinacrine IC_50_ concentrations were determined by cell death assays and compared to isogenic cell counterparts (Figure 1). For H2452, the quinacrine IC_50_ values decreased 55.8% from 3.46 ± 0.07 µM to 1.53 ± 0.16 µM in P452 cells, but there was no significant change between H2452 and C452 cells. For H226, the quinacrine IC_50_ values decreased 38.0% from 1.84 ± 0.12 µM to 1.14 ± 0.22 µM in P226 cells, but there was also no significant change between H226 and C226 cells. These data suggest pemetrexed-resistance cells had an enhanced response to quinacrine compared to native counterparts, but cisplatin resistance did not alter quinacrine efficacy. To our surprise, H226 (also P226 and C226) had a lower quinacrine IC_50_ concentration than H2452 and other cancer cell lines in prior studies (Preet et al., 2012; Park et al., 2018) including studies from our lab (Khurana et al., 2015; Kalogera et al., 2017; Jung et al., 2018). Previously reported quinacrine response biomarkers of p53 stabilization (Gurova et al., 2005) and PARP cleavage (Mohapatra et al., 2012) showed protein changes at 2.5 and 5 µM concentrations, with H226 being slightly more sensitive (Supplementary Figure 3).

The combination of quinacrine and cisplatin was also compared to cisplatin monotherapy (Figure 1B). The addition of relatively low-dose quinacrine enhanced the cytotoxicity of cisplatin in H2052, H226, and H2452 cell lines. The combination resulted in average combination index values from 0.68 to 0.75 (values less than 1 are considered synergistic), which can be interpreted as moderate synergism (values near 0.7) (Chou, 2010). Combining quinacrine with pemetrexed did not result in synergistic values (data not shown) Unexpectedly, H2052 cells were very sensitive to relatively low concentrations of quinacrine even in the absence of cisplatin.

### NF2-Mutated Cells Are More Sensitive to Quinacrine

**FIGURE 2:**
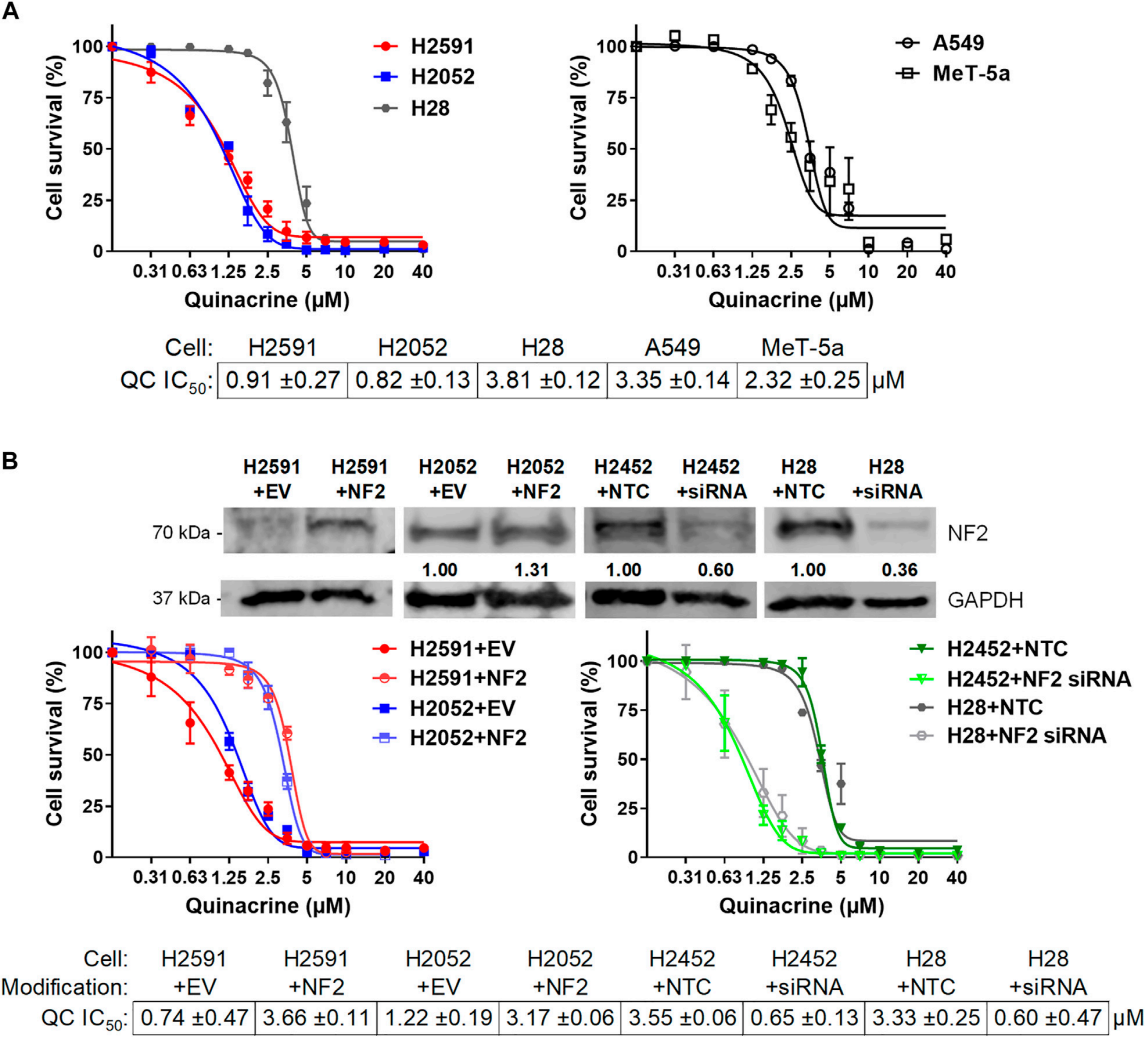
Quinacrine response changes based on NF2 status. **(A)** Cell death assays with quinacrine in NF2-mutated H2591 and H2052 cells compared to H28 **(*left*)** and A549 lung cancer cells and non-malignant MeT-5A mesothelium cells **(*right*)**; **(B)** Ectopic NF2 expression or NF2 knockdown in NF2-mutated or NF2-wildtype cells, respectively. Immunoblot validation of ectopic expression or knockdown was compared to cells with empty vector or non-targeting control transfections.

Based on the difference in quinacrine response between H226 and H2452 (Figure 1A), and also the sensitivity of H2052 to low doses of quinacrine (Figure 1B), we tested quinacrine across mesothelioma, non-malignant mesothelial, and lung cancer cell lines (Figure 2A) as well as ovarian and breast cancer cell lines that had been previously reported (data not shown). Although there were minor differences in quinacrine response among cell lines, the most dramatic decrease in quinacrine IC_50_ was seen in H2591 and H2052 (Figure 2A), and also H226 (Figure 1A). These three cell lines have NF2 mutations; H2591 has heterozygous deletion, H2052 has a mutation [also a LATS2 mutation (Murakami et al., 2011)], and H226 has a copy number deletion for the NF2 gene (Sekido et al., 1995). These data suggest cells with inactivating NF2 mutations may be more sensitive to quinacrine.

To confirm the role of NF2 mutations for quinacrine sensitivity, wildtype NF2 was ectopically expressed in NF2-mutated H2591 and H2052 cells and silenced in H2452 and H28 cells without NF2 mutations (Figure 2B). Wildtype NF2 expression in H2591 and H2052 resulted in 4.9- and 2.6-fold increases for quinacrine IC_50_ concentrations, respectively. For comparison, wildtype NF2 was also transfected in H2452 cells (containing endogenous wildtype NF2), which had no significant difference in quinacrine response (Supplementary Figure 4). The primary hippo pathway transcription factor YAP1 and expression target cyclin D1 were also decreased with ectopic NF2 expression for H2591, suggesting an increase in active NF2 (Supplemental Figure 5). In contrast, silencing NF2 expression in H2452 and H28 resulted in an 81.7 and 82.0% decrease for quinacrine IC_50_ concentrations, respectively (Figure 2B, right). These data suggest that the NF2 status of a cells can regulate the response to quinacrine.

### Quinacrine Induces a Different Genetic Expression Response in NF2 Mutant Cells

**FIGURE 3:**
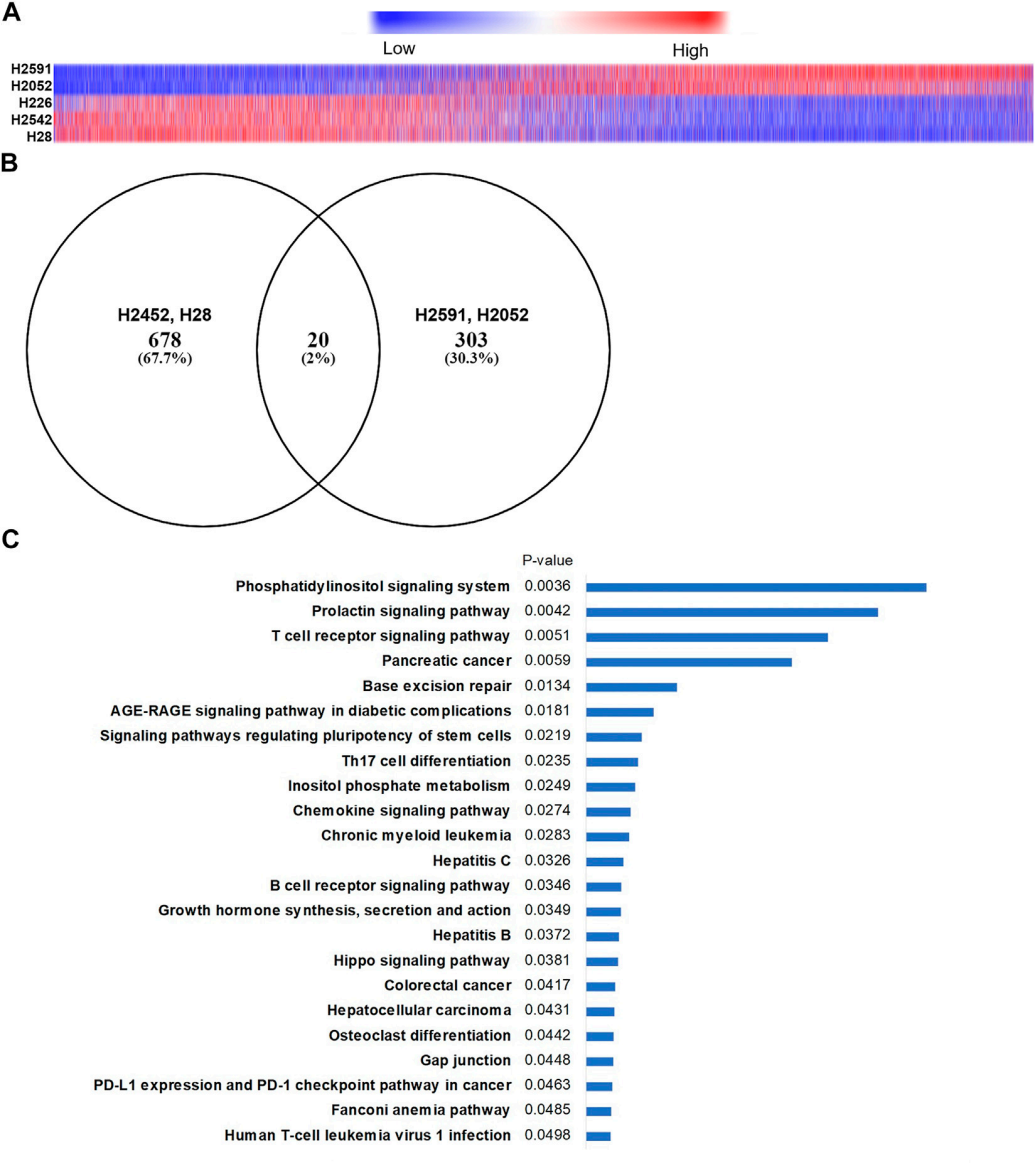
RNA sequencing in mesothelioma cells with and without NF2 mutations. **(A)** Heatmap of 17,000 genes identified (has Ensembl annotation, median expression ≠ 0) showing differential expression changes after 6 h quinacrine treatment compared to vehicle. Order is based on H2591 expression changes. Approximately 3,000 genes had a significantly different (*p* < 0.05) response from quinacrine treatment when comparing H2591/H2052 NF2-mutated cells to H2542/H28 NF2 WT cells (see Supplementary Figure 6 for pathway comparison); **(B)** Venn diagram of differential expressed genes for quinacrine-treated cells compared to untreated cells. **(C)** Top related KEGG 2021 pathways of quinacrine-induced genetic expression changes for H2591 and H2052 compared to vehicle (determined using Enrichr).

Prior studies on the anticancer mechanism of action for quinacrine suggest many pathways may be regulated (Oien et al., 2019), but none of these reported pathways are clearly associated with NF2 signaling. To address the mechanism of action for quinacrine in NF2-mutated cells, RNA sequencing was done on five cell lines treated with quinacrine or vehicle (Figure 3). When arranged by expression changes compared to vehicle, the quinacrine responses of NF2 mutant H2591 and H2052 cell lines were markedly different from the response of H2452 and H28 (Figure 3A). H226 primarily clustered with H2542 and H28 expression changes. Since H226 only has an NF2 copy number deletion and the biological effect of this alteration was not clear, this cell line was omitted from most of the remaining studies. To identify pathways altered in H2591 and H2052 cells, significant changes between quinacrine treatment and vehicle were identified and compared to the same analysis for H2452 and H28 cells (Figure 3B). Comparison of these expression changes to the KEGG 2021 pathway database for the 303 genes with quinacrine-induced expression changes exclusive to H2591 and H2052 resulted in significant similarity to 23 pathways (Figure 3C). Note that this analysis does not provide evidence if the pathway was “turned on” or “turned off.” When comparing quinacrine-induced changes between H2591 and H2052 to H2452 and H28, 3168 genes had differential expression and these changes aligned with >50 KEGG pathways (Supplementary Figure 4A). Gene expression changes induced by quinacrine for H2591 and H2052 aligned more with pathways for hippo, TGFß, cell cycle, and reactive oxygen species stress (Supplementary Figure 4B). In contrast, quinacrine-induced changes to H2452 and H28 included with autophagy and DNA repair that have been previously reported (Oien et al., 2019). Both comparisons provide supporting evidence that the hippo pathway and closely-associated signaling is perturbed by quinacrine in cells with inactivating NF2 mutations. These lack of overlapping changes (Figures 3A,B) also suggest that quinacrine may only have a limited effect on the pathways related to previously reported cytotoxic mechanisms for H2591 and H2052 cells, although this suggestion was not confirmed.

### Quinacrine Modulates Hippo-Related Signaling in NF2 Mutant Cells

**FIGURE 4:**
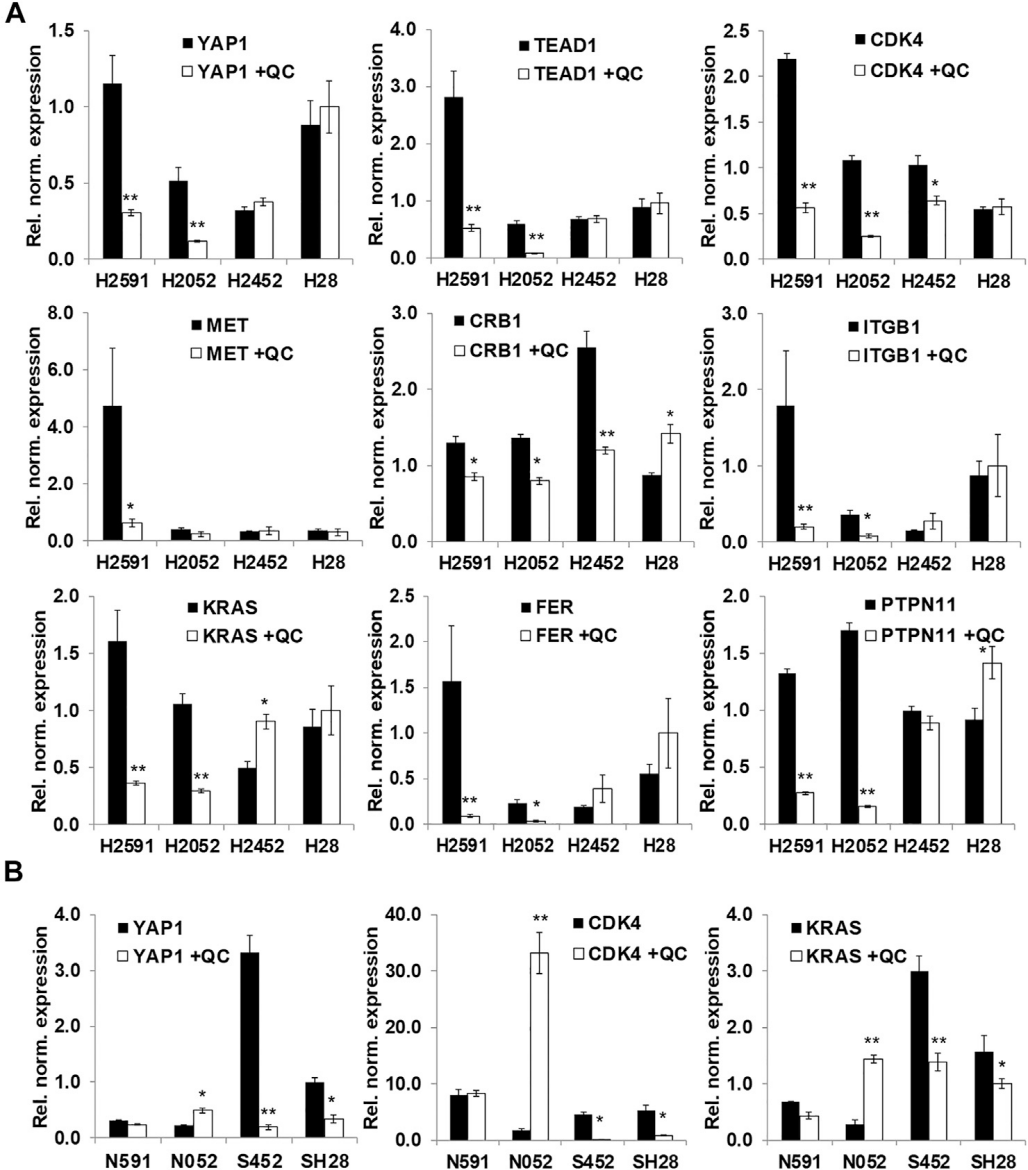
Validation of RNA sequencing results with a focus on the hippo pathway. **(A)** qPCR validation of selected genes with quinacrine (QC, 5 µM for 6 h); **(B)** Quinacrine-induced mRNA changes with NF2 ectopic expression (N591 and N052 represent H2591 and H2052 with NF2 expression, respectively) and siRNA knockdown of NF2 (S452 and SH28 represent H2452 and H28 with NF2 shRNA, respectively). Values represent expression normalized to RPLP0. *p < 0.05, **p < 0.005.

**FIGURE 5:**
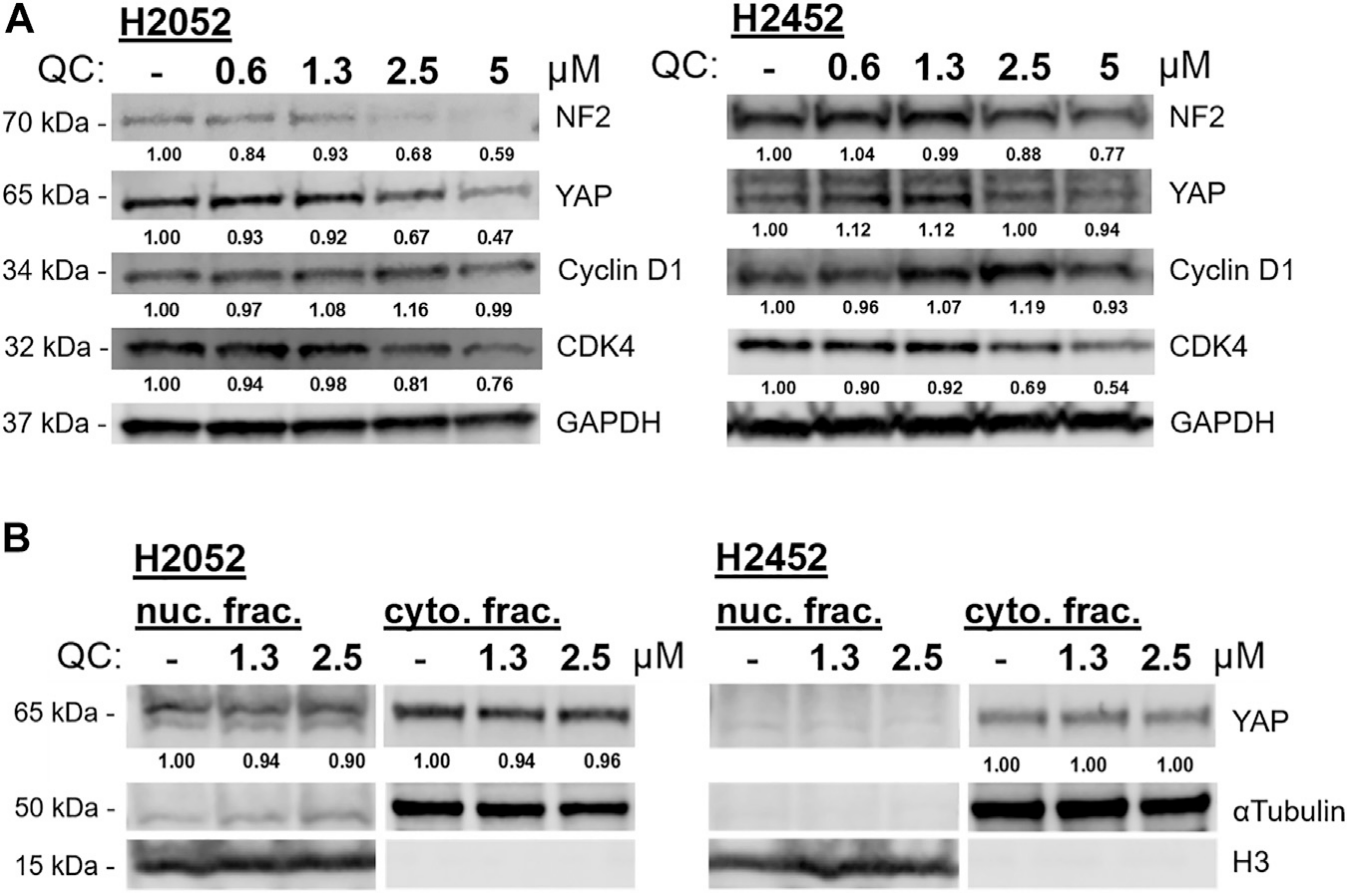
**(A)** Quinacrine (QC)-induced protein changes to biomarkers of the hippo signaling pathway at incrementing concentrations. **(B)** Nuclear and cytoplasmic fractions for YAP detection with quinacrine treatment. Densitometry values are normalized to respective loading control (GAPDH, αtubulin, or H3).

Based on RNA sequencing results, several genes expression changes from quinacrine treatment were validated (Figure 4) with a focus on the hippo pathway. The hippo pathway was identified in both KEGG pathway database comparisons (Figure 3C for H2591 and H2052 with quinacrine compared to vehicle, Supplementary Figure 4A for quinacrine-induced changes between NF2 mutant and wildtype cells). Several genes for H2591 and H28 had significantly decreased expression from quinacrine treatment while gene expression for H2452 and H28 remained nonsignificant or increased (Figure 4A; *YAP1*, *TEAD1*, *ITGB1*, *KRAS*, *FER*, *PTPN11*). Ectopic expression and knockdown of NF2 altered the expression and response to quinacrine for *YAP1*, *CDK4*, and *KRAS* (Figure 4B), and also in pemetrexed-resistant P226 compared to H226 cells (Supplementary Figure 7). The canonical hippo transcription factor YAP decreased in response to quinacrine at the protein level for H2052 and H2452 cells, but only at higher concentrations (≥2.5 µM) for H2452 cells (Figure 5A). Quinacrine-induced YAP changes were similar between nuclear and cytoplasmic protein fractions (Figure 5B), which suggests quinacrine may be regulating YAP expression. However, NF2 wildtype H2452 and H28 (and H2052 with ectopic wildtype NF2 expression) do not display reduced *YAP* mRNA in response to quinacrine, which suggests the quinacrine mechanism of action may be different in NF2 wildtype cells.

### Quinacrine Promotes G1 Arrest in Cells With *NF2* Genetic Alteration

**FIGURE 6:**
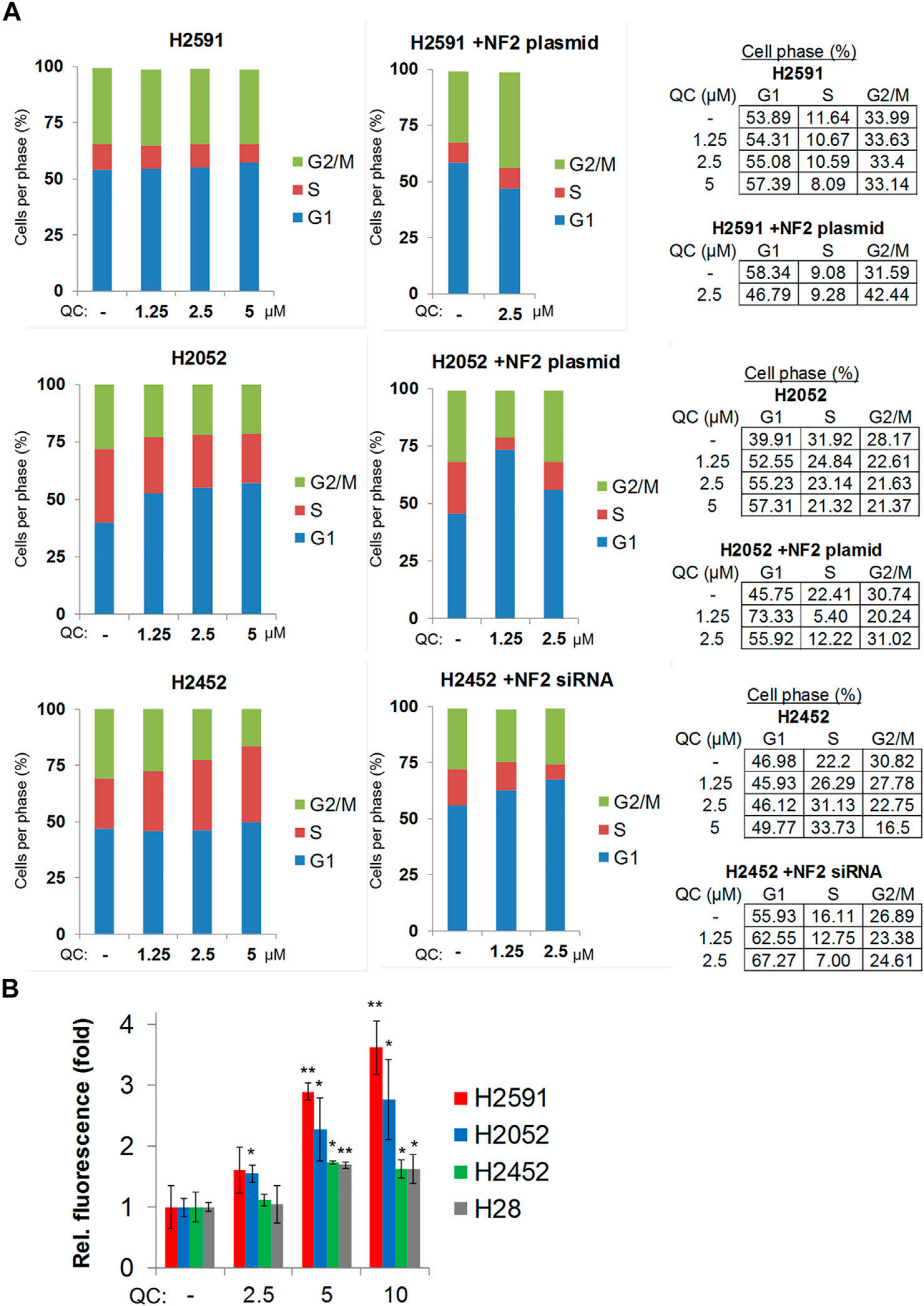
Quinacrine induces a G1-phase cell cycle arrest and increased reactive oxygen species in mesothelioma cells with inactivating NF2 mutations. **(A)** Cell cycle analysis; **(B)** Total reactive oxygen species detection.

Hippo signaling regulates many pathways including cell proliferation, cell attachment, and cell cycle (Moroishi et al., 2015). Based on our observed changes of cyclin D1 and CDK4 in response to quinacrine (Figure 5), we profiled the effects of quinacrine on cell cycle for cells with inactivating NF2 mutations (Figure 6A). Prior studies have reported that quinacrine causes an S-phase cell cycle arrest (Preet et al., 2012). We also observe an S-phase arrest when quinacrine is applied to H2542 cells, but the fraction of cells in the G1 phase is increased after quinacrine treatment in H2591 and H2052 cells lines (Figure 6A). Furthermore, the quinacrine-induced distributions can be disrupted by NF2 ectopic expression or siRNA knockdown of H2591/H2052 and H2452, respectively, although NF2 ectopic expression increased cells in the G2/M phase. The pathway analyses based on RNA sequencing data also indicated that reactive oxygen species and cellular stress pathways maybe have a different response to quinacrine between H2591/H2052 and H2452/H28 cells. Short-term (4 h) quinacrine exposure increased total reactive oxygen species in all 4 cell lines at 5 and 10 µM, but the largest increases were in H2591 and H2052 cells (Figure 6B). Overall, these data suggest cells have a different response to quinacrine based on the presence of active or mutated NF2.

## Discussion

The FDA approved drug treatments for malignant pleural mesothelioma are a combination of pemetrexed with cisplatin and the recently FDA- and EMA-approved antibody combination of nivolumab and ipilimumab. Unfortunately, there are few other emerging drug therapies for mesothelioma and very limited experimental drugs targeted at frequent mutations of mesothelioma (Oien et al., 2020). Of the few studies on targeted drugs that include mesothelioma, most late-stage studies are focused on BAP1 mutations while inactivating NF2 mutations (and other hippo pathway mutations) are more prevalent in pleural mesothelioma patients. Moreover, NF2 has the overall lowest expression in mesothelioma compared to all other TGCA datasets (Supplementary Figure 8). Here, we initially describe the potential of quinacrine as an adjuvant therapy to cisplatin or pemetrexed and the response of cisplatin- or pemetrexed-resistant cells to quinacrine monotherapy. Most importantly, we reveal that mesothelioma cells with NF2 inactivating mutations are more sensitive to quinacrine.

Our prior studies of quinacrine in gynecologic cancers revealed that drug-resistant cells and xenografts had an enhanced cytotoxic response to quinacrine (Khurana et al., 2015; Kalogera et al., 2017; Thirusangu et al., 2021). In ovarian cancer cells, cells with artificially-generated drug resistance were more susceptible than isogeneic cells without artificial drug resistance (Khurana et al., 2015). Combining quinacrine with cisplatin *in vitro* or carboplatin *in vivo* also increased cell and tumor death, respectively. In a similar study, endometrial cancer cells that were the least susceptible to cisplatin alone had the highest response benefit when quinacrine was added in combination (Kalogera et al., 2017). In this study, new resistant cells lines were generated and we found that pemetrexed-resistant P452 and P226 cell lines were more susceptible to quinacrine, but not C452 and C226 (Figure 1A). However, the addition of quinacrine to cisplatin resulted in a moderately synergistic response for H2452 and H226 cells (and some combinations of H2052; Figure 1B). After these initial studies and when we migrated our focus to the hippo pathway, we found that *YAP1* and *CDK4* mRNA expression increased in P226 cells compared to H226 cells (Supplementary Figure 7). The hippo pathway and increased YAP signaling are emerging as potential drivers for resistance to cancer therapy (Nguyen and Yi, 2019). The question of whether or not quinacrine could be inhibiting YAP that may be driving resistance in multiple cancer subtypes (including other resistant mesothelioma cells) was beyond of the scope for this project.

Quinacrine has already been used clinically as an antimicrobial drug with daily doses as high as 2,000 mg (Dubois, 1954; Engeset, 1958). Quinacrine can accumulate in cells with increased tissue concentrations after repeated doses (Gayrard et al., 2005). However, clinical efficacy for quinacrine monotherapy in cancer patients has not been reported. To preclinically assess quinacrine at lower concentrations, most quinacrine studies focus on targeting susceptible tumors or using combination therapies [e.g. a completed Phase I trial in combination with erlotinib for nonsmall cell lung cancer (Bhateja et al., 2018)] (Oien et al., 2019). In this study, we demonstrate that mesothelioma cells with inactivating NF2 mutations are more susceptible to quinacrine *in vitro*, but the clinical efficacy for mesothelioma tumors with inactivating NF2 mutations will need to be assessed.

Targeting YAP and associated proteins for cancer is not a novel approach (Oien et al., 2020), but YAP and TEAD transcription factors, like many transcription factors, are often considered undruggable. Quinacrine decreases *YAP* expression in NF2 mutant cells (Figure 4A), although the specific mechanism of action remains to be determined. Tang *et al* recently identified preclinical TEAD inhibitors for NF2-deficient mesothelioma, although toxicity studies have not been reported for these compounds (Tang et al., 2021). The preclinical success of TEAD inhibitors compliments our findings and highlights the potential benefits for repurposed drugs.

A recent report was published with preclinical evidence supporting the use of quinacrine in pleural mesothelioma, but without any focus on NF2 mutations (Kulkarni et al., 2020). This report suggested the potential mechanism of action for quinacrine autophagy inhibition and apoptotic induction. The mechanistic studies mainly used H2452 and other cells without NF2 mutations for mechanistic studies. Regulation of autophagy and apoptosis was also reported in our prior ovarian cancer studies with quinacrine (Khurana et al., 2015; Jung et al., 2018), and our RNA sequencing data for H2452 and H28 (Figure 3) are also in line with these past reports.

The unexpected discovery that mesothelioma cells with inactivating NF2 mutations are more sensitive to quinacrine may support clinical indications for the repurposing of quinacrine as an anticancer agent. Our data are strengthened by increased quinacrine sensitivity in cell lines after NF2 siRNA knockdown (and the opposite effect for NF2 ectopic expression in NF2 mutant cells; Figure 2). Some genes transcripts were evaluated in NF2 ectopic expression and knockdown cells (Figure 4B), but it is unknown if these genetic alterations would lead to similar expression changes (clustering) as H2591 and H2052 cells when treated with quinacrine. Quinacrine-induced differential expression for H226 cells clustered with H2452 and H28 (Figure 3), which suggests that an *NF2* copy number deletion may affect the drug sensitivity (Figure 1) without matching the quinacrine-induced expression signature of H2591 and H2052 cells. The direct target of quinacrine that regulates the YAP and hippo pathway is not yet known, and our RNA sequencing data (including H226) will support future in-depth inquires to understand a specific mechanism of action.

To our knowledge, this is the first study focusing on the use of quinacrine for cancer cells with inactivating NF2 mutations in mesothelioma. Further inquiry provides evidence that cells with NF2 mutations are more sensitive to quinacrine through changes in the hippo pathway, although any related direct target of quinacrine remains unknown. We have also generated drug-resistant mesothelioma cells and found that quinacrine sensitivity is enhanced in P452 and P226 pemetrexed resistant cells. Moreover, quinacrine enhances the cytotoxic effects of cisplatin when used in combination for mesothelioma cells. In summary, we report that quinacrine has repurposing potential for mesothelioma with NF2 mutations or chemotherapy resistance, and as an adjuvant treatment.

## Data Availability

The original contributions presented in the study are publicly available. This data can be found in the European Nucleotide Archive (ebi.ac.uk) under accession number PRJEB47449.
