# Supplementary material for: Quinacrine Has Preferential Anticancer Effects on Mesothelioma Cells With Inactivating NF2 Mutations
Source: Front Pharmacol. 2021 Sep 21;12:750352. doi: 10.3389/fphar.2021.750352 (PMC8490927; doi:10.3389/fphar.2021.750352)
Supplement: Supplementary file 1 [file DataSheet1.docx]

Supplementary Material

# Supplementary Tables

**Supplementary Table 1.** Cell line status of common mesothelioma genetic alterations. H2052 also has a LATS2 homozygous deletion. AE17 is a mouse (ms) cell line and A549 is a nonsmall cell lung cancer (LC) cell line.

| **Gene** | **Forward primer** | **Reverse primer** |
| --- | --- | --- |
| CDK4 | TGT CAA TGT AAC CCA AGG CTG | TCT ACA TGC TCA AAC ACC AGG |
| CRB1 | TGT CAA TGT AAC CCA AGG CTG | TCA CAG GCT TTC CCA CTT G |
| FER | GGC ACA GCT CCA TCA GAA TC | TGA CAA GAC TGG TTA TCT GGC |
| GNL3 | GAA CAA AGC CAA GTC GGG | GTC CAC TCT GGA CAA TGG |
| ITGA5 | TGC CTC CCT CAC CAT CTT | TGC TTC TGC CAG TCC AGC |
| ITGB1 | GGA TTC TCC AGA AGG TGG TTT CG | TGC CAC CAA GTT TCC CAT CTC C |
| KRAS | GGA GTA CAG TGC AAT GAG GG | CCA TAG GTA CAT CTT CAG AGT CC |
| LATS1 | GAA GCC ATT AGA GCG GAG AG | AGA ATA ACA CCA ACA CTC CAC C |
| LATS2 | AAC TCA CAG ATT TCG GCC TC | ACA CCG ACA GTT AGA CAC ATC |
| MET | ACA GTG GCA TGT CAA CAT CGC TCT AAT TCA GAG | GCT TCC ACT CTA TAT TTA GCT CGC TGT TC |
| PTPN11 | ACG GCA AGT CTA AAG TGA CC | ACT GTA CCC AAT GTT TCC ACC |
| TEAD1 | GCT CCA TTG GCA CAA CCA AG | ATG CCC AAT GTG CAC GAA GA |
| YAP1 | CCC AGA TGA CTT CCT GAA CAG | CCA TCT CCT TCC AGT GTT CC |
| YWHAZ | CTA CCG TTA CTT GGC TGA GG | CCA GTC TGA TAG GAT GTG TTG G |

**Supplementary Table 2.** Primers used for RT-qPCR.

# Supplementary Figures

**Supplementary Figure 1.** Artificially generated pemetrexed and cisplatin resistance in pleural mesothelioma cell lines. Clonogenic assays evaluation at the end of cyclic dosing for: (**a**) Pemetrexed-resistant (P452 and P226) compared to H2452 and H226, respectively; (**b**) Cisplatin-resistant (C452 and C226) compared to H2452 and H226, respectively. Error bars represent the standard error of 3 experiments, with representative plate images shown below.


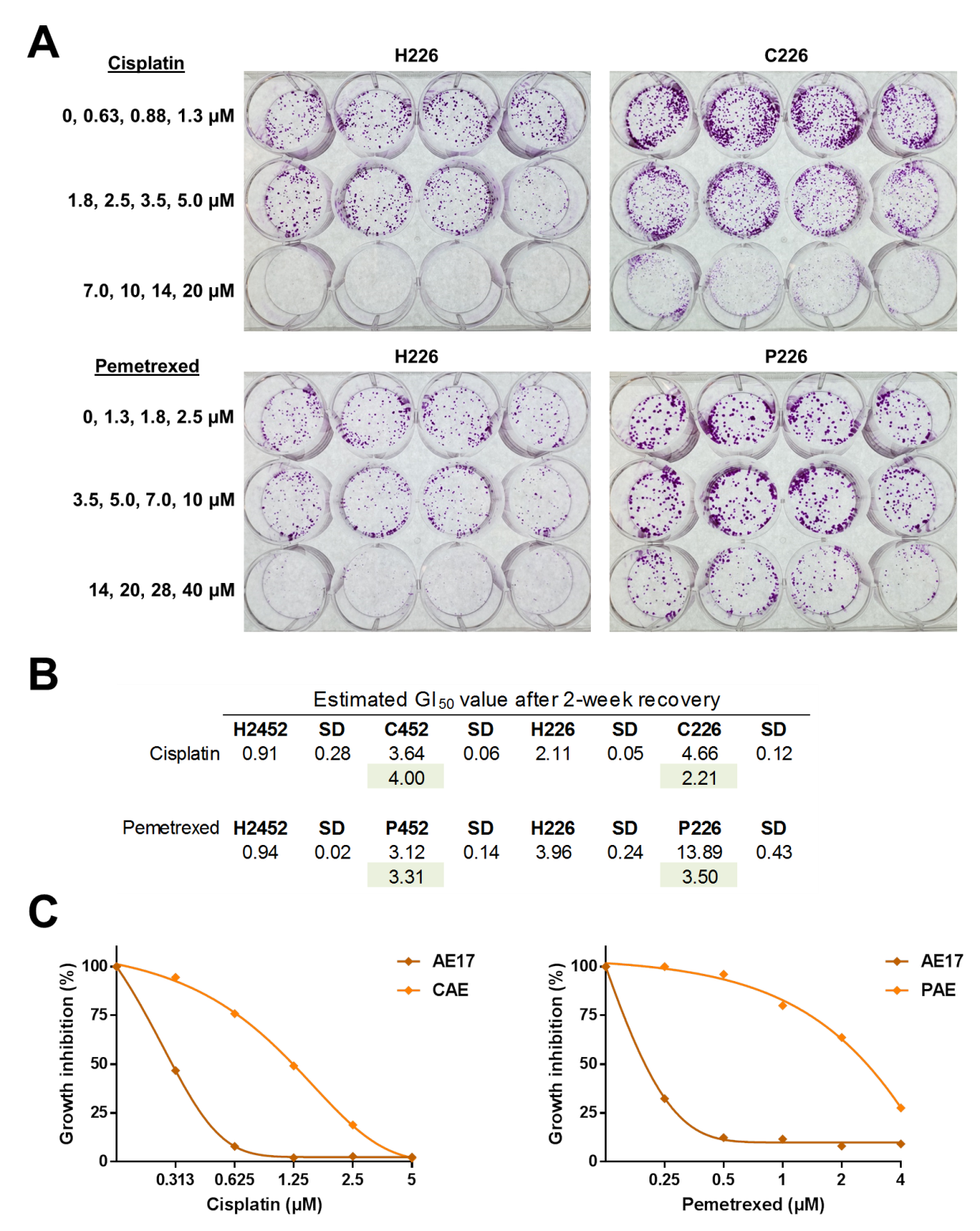


**Supplementary Figure 2.** Growth inhibition of chemotherapy resistant AE17 mouse cell line. Cell lines developed chemotherapy resistance *in vitro* by cyclic exposure to cisplatin or pemetrexed. Growth inhibition of parental AE17 mouse cells with cisplatin (*left*) or pemetrexed (*right*) resistant cells by clonogenic assay after cells were removed from drug for 2 weeks.

**
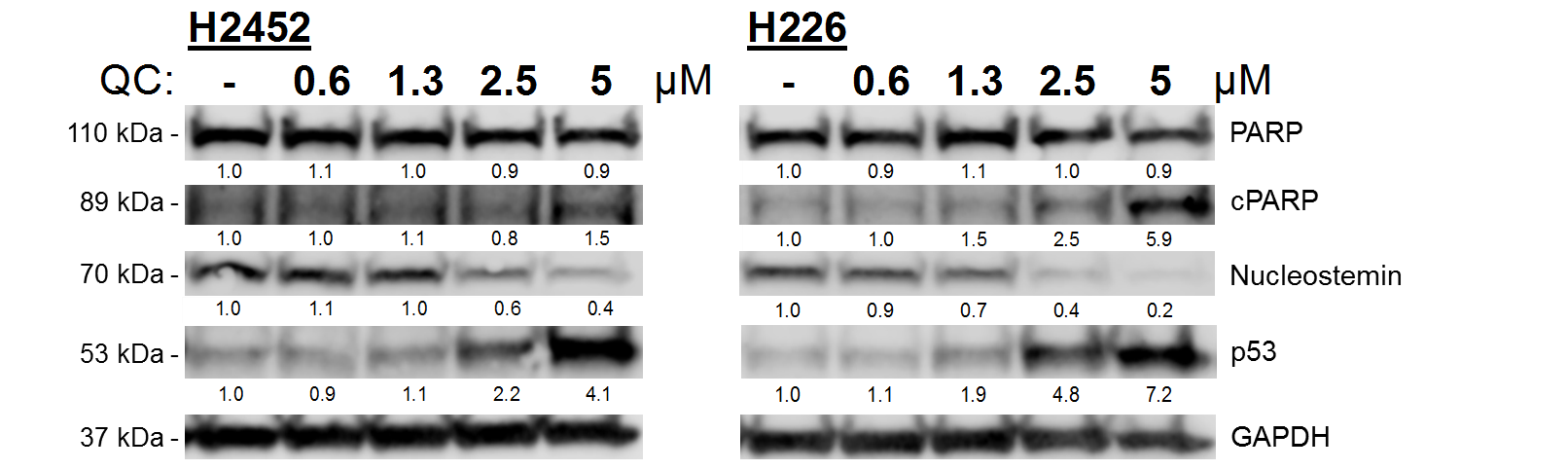
**

**Supplementary Figure 3.** Quinacrine increases p53 and cleaved PARP (*cPARP*) at different concentrations. Nucleostemin is a nucleolar protein that interacts with and negatively regulates p53. Cells were treated with quinacrine (*QC*) for 24 h. Densitometry values relative to GAPDH control.

|  | 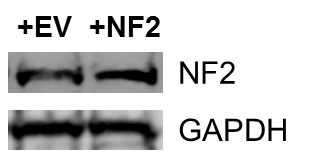 |
| --- | --- |

**Supplementary Figure 4.** NF2 ectopic expression in *NF2* WT cells does not significantly alter quinacrine cytotoxicity. Quinacrine (*QC*) 72 h IC_50_ is 3.35 µM for empty vector (*EV*) and 3.08 µM for ectopic expression (*NF2*). NF2 expression verified by immunoblot (*right*). Error bars represent SEM; n = 3.

**Supplementary Figure 5.** NF2 ectopic expression in H2591 cells alters NF2/hippo pathway signaling proteins. NF2 and loading control also shown in main Fig 2. Cells were treated with 800 µg/mL G418 selection agent for 1 week prior to obtaining cell lysates.

**A**

**
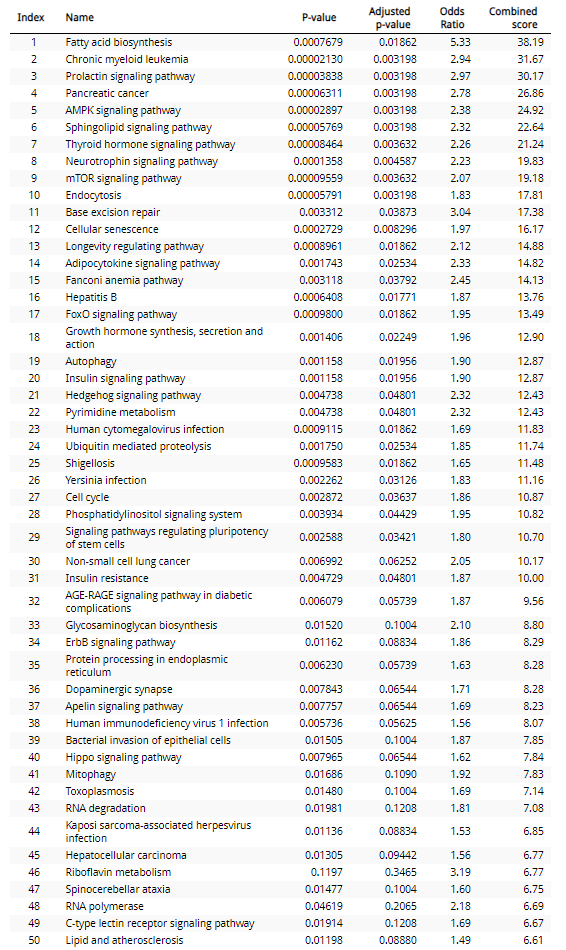
**

**B**

**Supplementary Figure 6.** Top pathway changes and differential expression of selected genes. **A.** Top 50 related pathways of quinacrine-induced genetic expression changes for H2591 and H2052 compared to H2452 and H28 cells. **B.** Differential expression values from RNA sequencing analysis for selected NF2/hippo/cell growth control signaling genes. Values represent mRNA normalized Log2 expression fold change from 5 µM quinacrine to untreated cells.

**Supplementary Figure 7.** Additional expression analyzed by RT-qPCR. Relative normalized expression for H226 and artificially generated pemetrexed resistant H226 (P226) with quinacrine (QC). Box shows *CDK4* with small y axis. n = 3, **p* < 0.05, ***p* < 0.005.


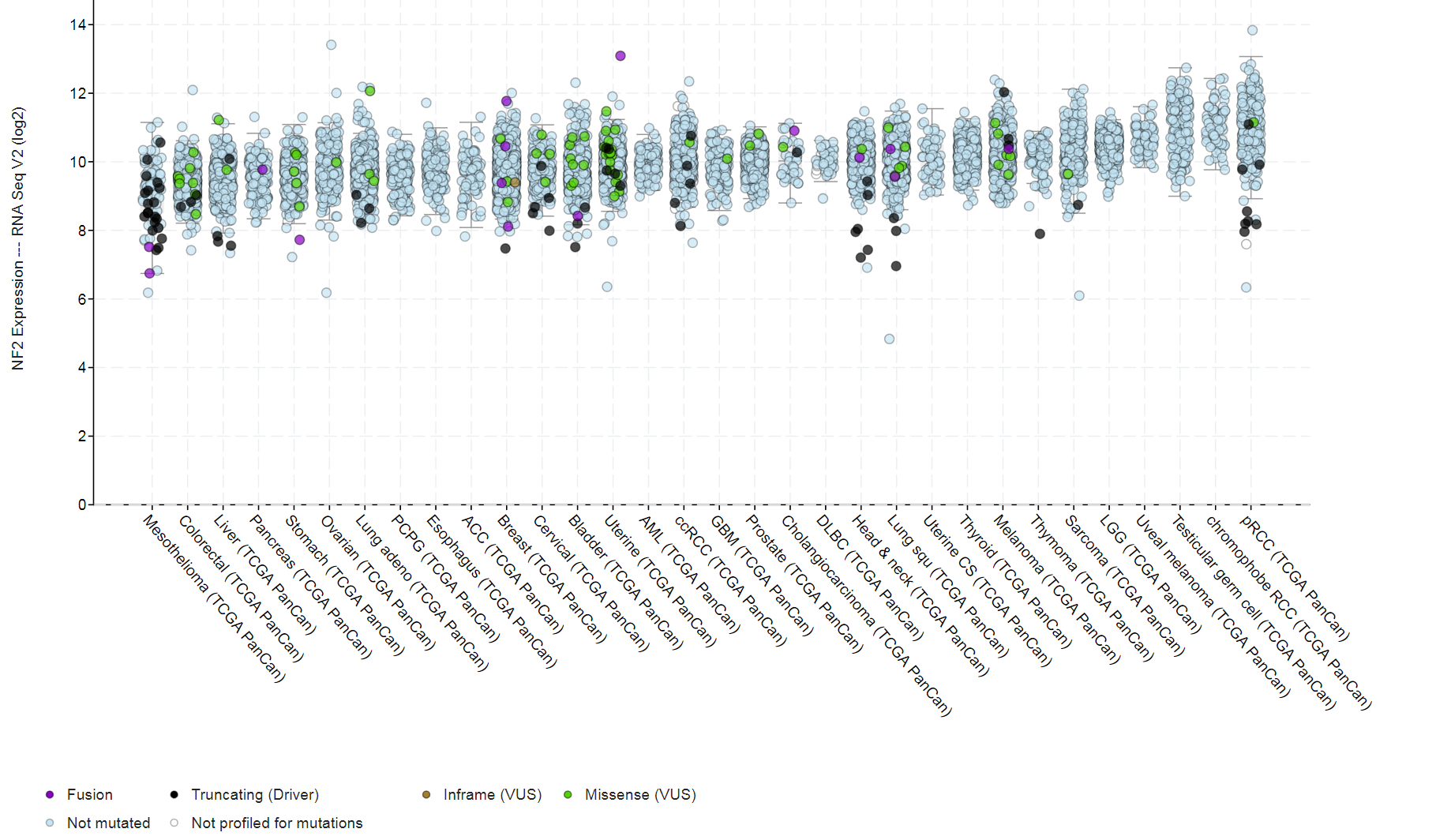


**Supplementary Figure 8.** *NF2* RNA expression of the 32 TCGA cancer datasets in order from lowest to highest median value. Graph generated at cbioportal.org.
